# Nutrient supplementation by genome-eroded *Burkholderia* symbionts of scale insects

**DOI:** 10.1038/s41396-023-01528-4

**Published:** 2023-10-13

**Authors:** Anna Michalik, Eugen Bauer, Teresa Szklarzewicz, Martin Kaltenpoth

**Affiliations:** 1 Department of Developmental Biology and Morphology of Invertebrates, Institute of Zoology and Biomedical Research, Faculty of Biology, Jagiellonian University, Krakow, Poland; 2 Department for Evolutionary Ecology, Institute for Organismic and Molecular Evolution, Johannes Gutenberg University Mainz, Mainz, Germany; 3 Department of Insect Symbiosis, Max Planck Institute for Chemical Ecology, Jena, Germany

**Keywords:** Microbial ecology, Metagenomics

## Abstract

Hemipterans are known as hosts to bacterial or fungal symbionts that supplement their unbalanced diet with essential nutrients. Among them, scale insects (Coccomorpha) are characterized by a particularly large diversity of symbiotic systems. Here, using microscopic and genomic approaches, we functionally characterized the symbionts of two scale insects belonging to the Eriococcidae family, *Acanthococcus aceris* and *Gossyparia spuria*. These species host *Burkholderia* bacteria that are localized in the cytoplasm of the fat body cells. Metagenome sequencing revealed very similar and highly reduced genomes (<900KBp) with a low GC content (~38%), making them the smallest and most AT-biased *Burkholderia* genomes yet sequenced. In their eroded genomes, both symbionts retain biosynthetic pathways for the essential amino acids leucine, isoleucine, valine, threonine, lysine, arginine, histidine, phenylalanine, and precursors for the semi-essential amino acid tyrosine, as well as the cobalamin-dependent methionine synthase MetH. A tryptophan biosynthesis pathway is conserved in the symbiont of *G. spuria*, but appeared pseudogenized in *A. aceris*, suggesting differential availability of tryptophan in the two host species’ diets. In addition to the pathways for essential amino acid biosynthesis, both symbionts maintain biosynthetic pathways for multiple cofactors, including riboflavin, cobalamin, thiamine, and folate. The localization of *Burkholderia* symbionts and their genome traits indicate that the symbiosis between *Burkholderia* and eriococcids is younger than other hemipteran symbioses, but is functionally convergent. Our results add to the emerging picture of dynamic symbiont replacements in sap-sucking Hemiptera and highlight *Burkholderia* as widespread and versatile intra- and extracellular symbionts of animals, plants, and fungi.

## Introduction

Many animals are intimately associated with microorganisms that affect various aspects of their biology, including nutrition, development, reproduction, and defense against natural enemies [1–3]. As such, symbiotic microorganisms play a fundamental role in the evolution of several groups of insects, promoting range expansions, speciation, and diversification. Insects feeding on plant sap deficient in essential amino acids and other nitrogenous compounds are particularly prone to engage in long-term symbiotic associations [3, 4]. In fact, mutualistic symbioses with microorganisms that supplement their unbalanced diet have allowed sap-feeding insects to adapt to such restricted food, expand into previously inaccessible ecological niches, and subsequently undergo adaptive diversification [5, 6].

Nutritional, heritable symbioses are ubiquitous within scale insects (Insecta, Coccomorpha)—a diverse group of plant sap-sucking hemipterans with economic importance, encompassing almost 8200 species [7]. They are widespread plant pests feeding on phloem sap and living on different parts of plants, including roots, branches, and leaves. Scale insects may be hosting both bacterial (e.g., *Tremblaya*, *Sodalis*, *Uzinura*, *Brownia, Burkholderia*) and fungal (*Ophiocordyceps*) nutritional symbionts, as well as facultative associates (such as *Rickettsia*, *Sphingomonas*, *Wolbachia*) whose functions for the hosts’ biology remain unknown [8, 9]. This large taxonomic diversity of microorganisms associated with scale insects, indicating multiple symbiont acquisition and replacement events, makes them a particularly interesting group for studying symbiosis evolution. Results of recent analyses with the use of molecular tools indicate that Flavobacteria (Bacteroidetes) represent the ancestral symbionts of scale insects, which during further evolution, have been lost in some lineages and replaced by other microorganisms that took over their function [8, 10].

The Eriococcidae (felt scales) is one of the neococcoid families, comprising about 560 species[11]. Its phylogeny and classification are still under discussion; however, the 18S rRNA-based phylogeny of scale insects suggests a paraphyletic origin of eriococcids [11, 12]. Previous studies showed that analyses of scale insect symbiotic microorganisms might help resolve relationships within and among Coccoidea families [13, 14]. Results of studies on symbionts of Eriococcidae, although few, also indicate their diversity [15–18]. Molecular studies revealed that some eriococcids live in mutualistic relationships with heritable bacteria *Arsenophonus*, *Kotejella* (in *Greenisca brachypodii*), and *Burkholderia* (in *Acanthococcus aceris* and *Gossyparia spuria*) [17, 18]. While the symbionts of *G. brachypodii* are localized intracellularly in specialized organs (bacteriomes), the *Burkholderia* symbionts of *A. aceris* and *G. spuria* stand out among obligatory, bacterial symbionts in hemipterans due to their occurrence in the cytosol of fat body cells, a localization that is reminiscent of the nitrogen-recycling *Blattabacterium* symbiont in cockroaches and some termites [19, 20]. Apart from the aforementioned bacterial symbionts, the occurrence of intracellular symbionts of fungal origin engaged in nitrogen recycling has been observed in a planthopper, *Nilaparvata lugens*, and Japanese cicadas [21, 22].

Bacteria belonging to the genus *Burkholderia s.l*. are widespread in nature and are also diverse regarding their lifestyle, environment, and ecological roles [23–25]. Some strains are pathogenic for plants (e.g., *Burkholderia gladioli* and *Burkholderia glumae*), humans, and other animals (e.g., *Burkholderia pseudomallei*) [24]. However, most *Burkholderia* species are common inhabitants of soil and rhizosphere with non-pathogenic influence on other organisms [26]. Some of them established beneficial, symbiotic associations with various eukaryotes including phytopathogenic and endophytic fungi, different plants, soil amebas, and insects [24, 25]. So far, within insects, mutualistic symbiotic relationships with *Burkholderia* were reported for phytophagous stinkbugs (Hemiptera, Pentatomomorpha), ants of the genus *Tetraponera* (Formicidae: Pseudomyrmecinae), Lagrinii beetles (Coleoptera, Tenebrionidae), and the two above-mentioned species of sap-feeding felt scale insects *A. aceris* and *G. spuria* (Eriococcidae) [17, 27–29]. In all of these insects except for the eriococcids, *Burkholderia* lives exclusively extracellularly in crypts or pouch-like organs associated with the gut (in stinkbugs and ants), or in accessory glands of the female reproductive system which produce a secretion that is deposited on the egg surface during oviposition, and in specialized cuticular structures in the larvae (in Lagriini beetles). In contrast, the scale insects *A. aceris* and *G. spuria* harbor *Burkholderia* in the cytoplasm of the fat body cells [17]. According to our knowledge, this is so far the only well-documented intracellular localization of *Burkholderia* symbionts in insects.

The functional role of *Burkholderia* symbionts for their host insects’ biology varies across different associations, including nutritional benefits, defense against pathogenic fungi and bacteria, resistance to insecticides, and nitrogen fixation [30–32]. The genome size of extracellularly localized *Burkholderia* symbionts ranges from 2.3–8.5 Mb and is generally smaller than the genomes of soil-living *Burkholderia* [31–33], exhibiting intermediate signs of genome reduction that are often observed in vertically transmitted intracellular and also extracellular symbionts [34–37]. However, information on genomic features and functional roles of intracellular *Burkholderia* symbionts remain lacking.

To fill this gap, we sequenced the genomes of the intracellularly localized *Burkholderia* symbionts of the two ericoccid species *A. aceris* and *G. spuria*. Our results show that *Burkholderia* strains associated with scale insects possess highly reduced genomes with few genes involved in housekeeping functions but retained genes responsible for the biosynthesis of essential amino acids and vitamins. These results reveal the smallest known genomes within the genus *Burkholderia* and uncover a case of a fat-body-localized symbiont functionally replacing bacteriome-localized symbionts in this group of insects.

## Materials and methods

### Insects

Adult females of maple felt scale*, A. aceris*, and European elm scale, *G. spuria*, were collected in Kraków in May and June 2013 and 2015 from branches of maples, *Acer platanoides* (Sapindaceae Juss.) and elms, *Ulmus laevis* Pall (Ulmaceae Mirb.), respectively. Both examined species are polyphagous insects feeding on phloem sap. Females of *A. aceris* live on trees (mainly from the family Sapindaceae), on rose bushes, and on boxwoods (http://scalenet.info/catalogue/Acanthococcus aceris/). Females of *G. spuria* live on trees (mainly from the family Ulmaceae), on mistletoes (*Viscum* spp.), and on grasses from the family Poaceae (http://scalenet.info/catalogue/Gossyparia spuria/).

### Histological and ultrastructural analyses

Adult females of *A. aceris* and *G. spuria* were fixed in 2.5% glutaraldehyde in 0.1 M phosphate buffer (pH 7.4) at 4 °C for a period of three months. The material was then rinsed in the same buffer with the addition of sucrose (5.8 g/100 ml) and postfixed in 1% osmium tetroxide for 1.5 h, dehydrated in a graded series of ethanol and acetone and embedded in epoxy resin Epon 812 (Serva, Heidelberg, Germany). Semithin sections (1 µm thick) were stained in 1% methylene blue in 1% borax, examined, and subsequently photographed under a Nikon Eclipse 80i light microscope (LM). Ultrathin sections (90 nm thick) were contrasted with uranyl acetate and lead citrate and examined and photographed under a Jeol JEM 2100 electron transmission microscope (TEM) at 80 kV.

### Symbiont localization by fluorescence in situ hybridization (FISH)

FISH was conducted with a *Burkholderia*-specific probe (Cy5-GCTCTTGCGTAGCAACTAAG). Females preserved in 100% ethanol were rehydrated, fixed in 4% formaldehyde, and dehydrated through incubations in 80%, 90%, and 100% ethanol and acetone. The material was then embedded in Technovit 8100 resin and cut into sections (1 µm thick). Hybridization was performed using a hybridization buffer containing: 1 M Tris–HCl (pH 8.0), 5 M NaCl, 20% SDS, 30% formamide, and distilled water. The slides were incubated in 200 μl of hybridization solution (hybridization buffer + 10 µM probe) overnight at room temperature [38]. Next, the slides were washed in PBS three times for 10 min, dried, and covered with ProLong Gold Antifade Reagent (Life Technologies). The hybridized slides were then examined using a confocal laser scanning microscope Zeiss Axio Observer LSM 710.

### DNA extraction and sequencing

Females of *A. aceris* and *G. spuria* destined for molecular analyses were preserved in 100% ethanol. The total genomic DNA was extracted from whole insects using Sherlock AX DNA Extraction kit (A&A Biotechnology) according to the manufacturer’s protocol. Isolated DNA was stored at −20 °C until further analyses. One pooled sample each of *A. aceris* and *G. spuria*, respectively, was sent to CeGat (Tübingen) for Illumina library preparation and sequencing. Paired-end libraries were prepared using the Nextera XT DNA Library Prep Kit (Illumina), and 2 × 150 bp PE reads were generated on a NovaSeq 6000 at sequencing depths of 9.8 Gb (*A. aceris*) and 26.0 Gb (*G. spuria*), respectively.

### Symbiont genome assembly and annotation

All computational analyses were performed using the KBase platform [39]. Sequence quality was assessed and trimmed with Trimmomatic v0.36 using default parameters [40]. Subsequently, sequencing reads were assembled to contigs with MEGAHIT v1.2.9 [41] for each insect species, respectively. To identify and filter sequences specific to the microbial symbionts, binning methods MetaBAT2 v1.7 [42] and MaxBin2 v2.2.4 [43] were used in combination with DASTool v1.1.2 [44] to cluster the contigs in several bins. The quality and taxonomic identity of those bins were then assessed with CheckM v1.0.18 [45]. For further analyses, the bins that were annotated as genus *Burkholderia* were used for both species. Since both extracted symbiont genomes were closely related to each other, the isolated symbiont genome of *A. aceris* was used as a reference to align and arrange the contigs of the *G. spuria* symbiont genome. Both symbiont genomes were then annotated using RAST [46], of which the annotated and called genes were used in further analyses.

### Phylogeny based on *Burkholderia* genomes

To check the taxonomic identity and phylogenetic context of the recovered *Burkholderia* symbionts, a phylogenetic tree was calculated by taking into account whole genome information. Potential *Burkholderia* reference species were selected based on a recent phylogenetic study of *Burkholderia* species [47]. These reference genomes were then annotated in KBase [39] with RAST [46]. Within KBase, a multiple sequence alignment was performed based on a conserved set of 49 COG (Clusters of Orthologous Groups) family domains, respectively. The alignments were concatenated, and an approximately maximum likelihood tree was calculated using FastTree2 v2.1.9 [48] with 1000 resamples for local branch support values. The resulting tree was then exported in Newick format and edited with MEGA11 v11.0.10 [49].

### Comparative genomics of related symbionts

To pinpoint the putative metabolic function of the recovered *Burkholderia* symbionts, their metabolic capabilities were put in context with other insect symbionts using a comparative genomics approach. For this, automatic gene annotations of the *Burkholderia* symbionts and other relevant hemipteran symbionts (Fig. S1) were assigned to COGs (Clusters of Orthologous Groups) within KBase [39]. Since most symbionts of our set are involved in co-enzyme and amino acid metabolism, genes of those metabolic categories were extracted and then compared based on their presence and absence in the different genomes. The results were then displayed in a heatmap using the R package ComplexHeatmap [50].

## Results

### Localization of *Burkholderia* symbionts in *A. aceris* and *G. spuria*

**Fig. 1:**
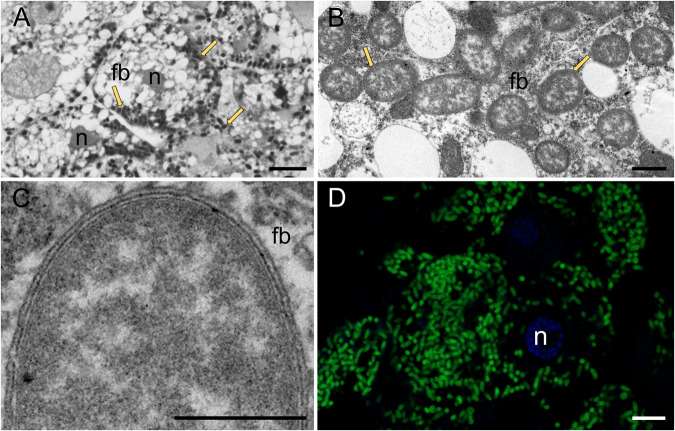
Intracellular localization of *Burkholderia* symbionts in the fat body cells of Eriococcidae. **A**–**D** *Burkholderia* cells in the cytoplasm of the fat body cells of *G.* *spuria*, fb—fat body, n—fat body cell nucleus, arrows—*Burkholderia* cells. **A** Light microscopy, scale bar—10 µm, **B**, **C** Transmission electron microscopy, scale bar—1 µm, **D** Confocal microscopy, green - *Burkholderia* cells, scale bar—10 µm.

As reported previously [17], rod-shaped *Burkholderia* bacteria occur intracellularly in the cytosol of fat body cells of *A. aceris* and *G. spuria* (Fig. 1A–C). The presence of high densities of symbiont cells in the fat body cells was confirmed by means of fluorescence in situ hybridization with a *Burkholderia*-specific probe (Fig. 1D). Transmission electron microscopy confirmed the presence of three membranes surrounding the *Burkholderia* cells (two bacterial membranes and one symbiosome membrane) (Fig. 1C), supporting previous observations describing the eriococcid symbiosis as the first documented intracellular association between insects and *Burkholderia* [17].

### Genome characteristics

Illumina paired-end sequencing of shotgun libraries from total DNA of *A. aceris* and *G. spuria* resulted in 139 and 177 million raw reads, respectively, that were quality checked and trimmed and subsequently assembled into 20,613 (11,843,469 bp) and 78,051 (264,520,377 bp) contigs using Megahit v1.2.9 [41] as implemented in KBase [39]. Binning of contigs with MetaBAT2 v1.7 [42] and MaxBin2 v2.2.4 [43] and combining the results with DASTool v1.1.2 [44] and subsequent reassembly in Geneious v. 2019.1.3 yielded three contigs with 866,825 bp taxonomically assigned to the genus *Burkholderia* for *A. aceris* and six for *G. spuria*. Synteny analyses with Clinker [51] and Nucmer [52] revealed a high degree of similarity and perfect synteny between the symbiont genomes of the two host species, based on which the *G. spuria* contigs could be easily arranged into three scaffolds by comparison with the *A. aceris* contigs. Repeat regions at the ends of the contigs and scaffolds prevented assembly into circular chromosomes.

**Fig. 2:**
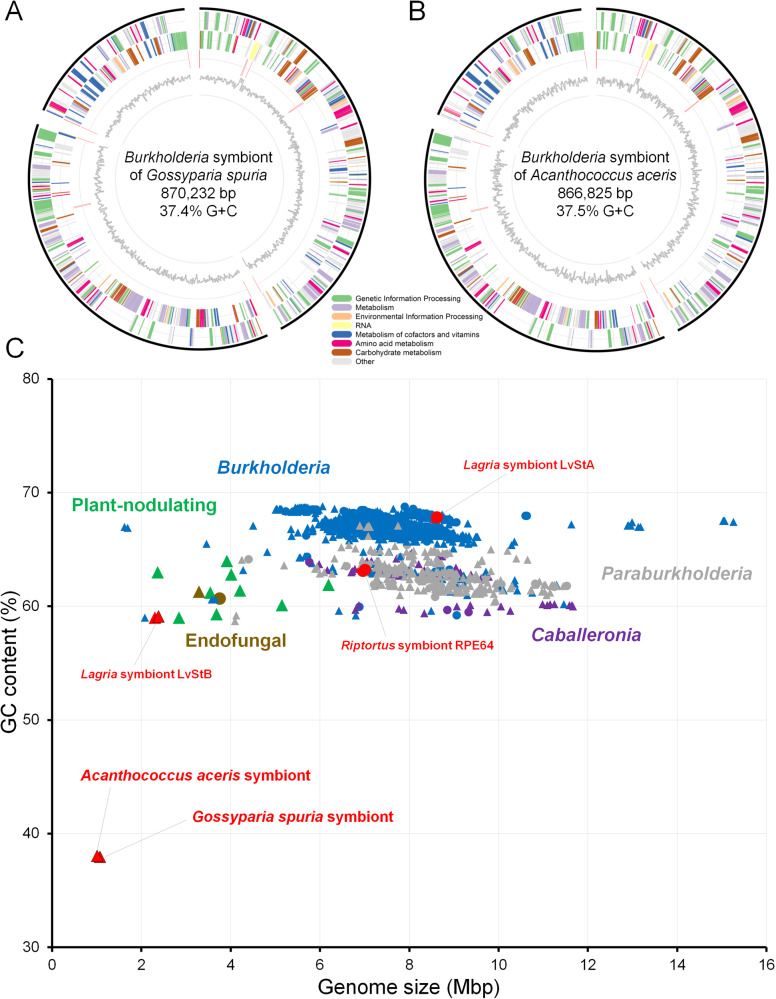
Genomic features of *Burkholderia* symbionts in Eriococcidae. **A**, **B** Circular diagrams of *Burkholderia* symbiont genomes from **A** *G. spuria* and **B** *A. aceris*. **C** Size and GC content plot for sequenced genomes of *Burkholderia sensu lato* strains, highlighting the Eriococcidae symbiont genomes as the smallest and most AT-biased genomes among all *Burkholderia s.l*. sequenced to date. Colors highlight genus-level affiliation (*Burkholderia*—blue, *Paraburkholderia*—gray, *Caballeroni*a—violet) and host associations (fungi—brown, plants—green, insects—red), respectively, and shapes denote draft (triangles) and complete genomes (circles).

The draft genome of the *G. spuria* symbiont exhibited a size of 870,232 bp, a G + C content of 37.4% and encoded for 726 CDS after RAST annotation [46] in KBase [39]. CheckM v1.0.18 [45] predicted a completeness of 42.4%, but the annotation of 39 tRNAs for all 20 amino acids indicates that the genome is in fact almost complete. Low estimates of completeness based on searches for conserved single-copy marker genes were expected, since the investigated symbionts have experienced genome erosion, resulting in the loss of many genes that are otherwise conserved across taxonomically related *Burkholderia* reference genomes. Similarly, the *A. aceris* symbiont draft genome was 866,825 bp in size, with a G + C content of 37.5%, 748 predicted CDS, an estimated completeness of 39.7%, and 38 tRNAs annotated for 19 amino acids. tRNA-Phe was missing in the original annotation but could be manually annotated based on the comparison with the *G. spuria* symbiont genome, resulting in 39 tRNAs for all 20 amino acids (Fig. 2). Furthermore, based on the coverage and binning approach, no other contigs could be found in the metagenome that would correspond to the genome of the symbionts. Additionally, the symbiont contigs were flanked by repeat regions (Fig. 2), which are generally difficult to sequence, further indicating that the recovered genomes are indeed complete.

### Functional potential of the *Burkholderia* symbionts in eriococcids

**Fig. 3:**
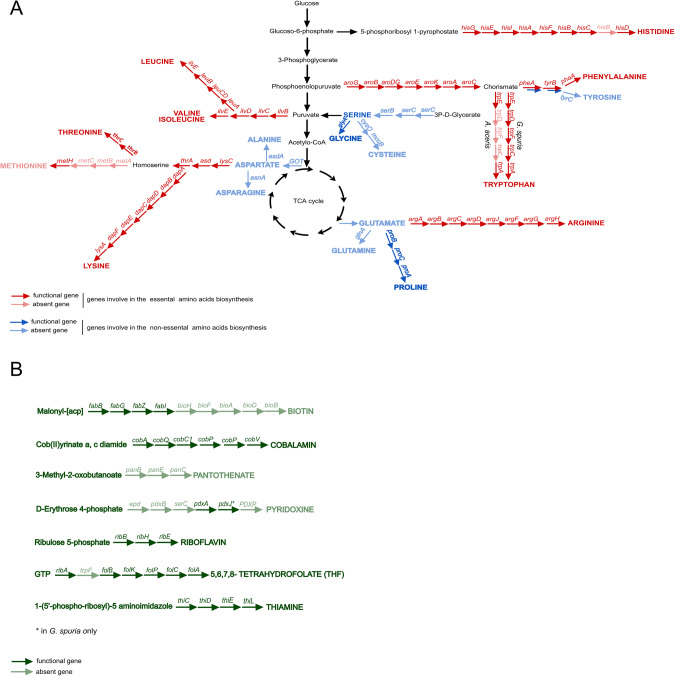
Nutritional potential of intracellular *Burkholderia* symbionts in Eriococcidae. Biosynthesis pathways of amino acids (A) and B vitamins (B) encoded in the genomes of *Burkholderia* Arrows represent individual enzymatic steps, with the abbreviated name of the gene shown above. Dark-colored arrows indicate the presence of the respective gene, light-colored arrows the absence.

The genomes of the two eriococcid symbionts show a high degree of similarity in their functional gene content (Fig. 3). Despite their severely reduced genomes, both retain complete biosynthetic pathways for the essential amino acids leucine, isoleucine, valine, threonine, lysine, arginine, histidine (except for *hisB*), and phenylalanine, as well as precursors for the semi-essential amino acid tyrosine. A complete biosynthetic pathway for tryptophan biosynthesis is encoded in the *G. spuria* symbiont genome, whereas *trpC, trpD*, and *trpF* were disrupted by frameshift mutations in the *A. aceris* symbiont genome. Both symbionts are lacking the methionine biosynthesis genes *metA, metB*, and *metC*, but encode the cobalamin-dependent methionine synthase MetH (Fig. 3A).

In addition to the pathways for essential amino acid biosynthesis, both symbionts appear to be capable of synthesizing multiple cofactors, including riboflavin (vitamin B2), cobalamin (vitamin B12), and folate (vitamin B9). The *G. spuria* symbiont additionally encodes pathways for heme and thiamine biosynthesis (vitamin B1) that are lacking from the *A. aceris* symbiont genome. Furthermore, the symbiont of *G. spuria*, but not that of *A. aceris*, encodes multiple transporters as well as enzymes involved in the uptake and assimilatory reduction of sulfate (not shown).

Comparisons with the functional potential of other hemipteran obligate and co-obligate symbionts reveal broad commonalities in essential amino acid biosynthetic capabilities (Fig. S1). By contrast, the distribution of B vitamin biosynthesis pathways across symbionts is patchier, with *Baumannia cicadellinicola* being the most versatile B vitamin producer. However, the symbionts of *A. aceris* and *G. spuria* stand out in their ability to synthesize cobalamin, a pathway that is lacking in most other hemipteran symbionts, with the exception of *Hodgkinia cicadicola* [53] and *Evansia muelleri* [54]. Concordantly, *Hodgkinia, Evansia*, and the *Burkholderia* symbionts of eriococcids all encode the cobalamin-dependent methionine synthase MetH, instead of the cobalamin-independent version MetE that is encoded by most other symbionts. Thus, it appears likely that cobalamin biosynthesis is only required to provide the cofactor for MetH.

### Phylogenetic affiliation of the eriococcid symbionts

**Fig. 4:**
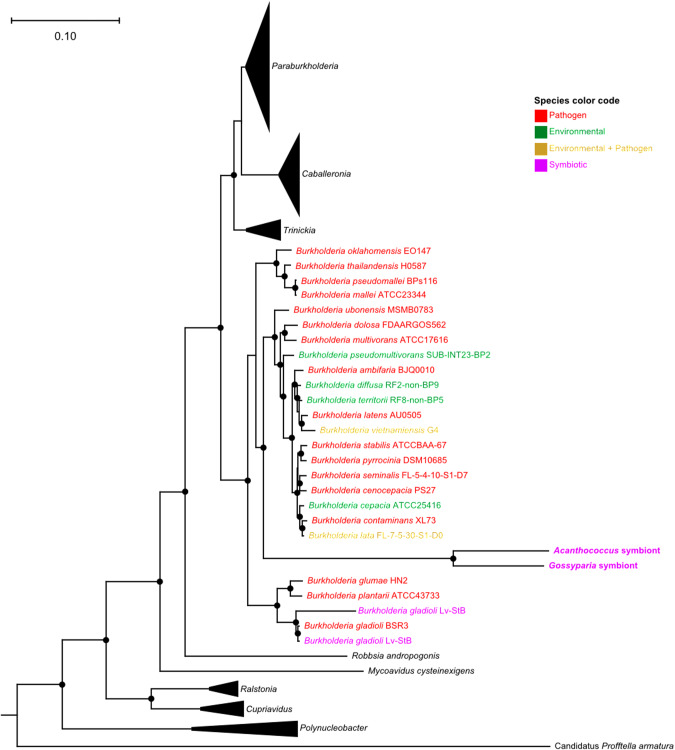
Phylogenomic placement of Eriococcidae symbionts within *Burkholderia s.str*. The phylogeny was constructed based on 49 COG family domains showing the relationships among *Burkholderia* strains. Different colors indicate ecological differences (environmental, pathogenic, symbiotic). Filled circles on the nodes represent branch support values above 90% from an approximately maximum likelihood reconstruction using FastTree2 v2.1.9 [48] with 1000 resamples.

Phylogenomic analyses of the *A. aceris* and *G. spuria* symbionts and other sequenced Burkholderiales based on 49 COG family domains reveal their affiliation with the genus *Burkholderia s.str*. (Fig. 4). This is insofar interesting as most other insect-associated *Burkholderia s.l*. fall into the genera *Caballeronia* and *Paraburkholderia* [25], with only the defensive symbionts of *Lagria* beetles [27, 31, 32] and some environmentally acquired gut symbionts in two hemipteran species *Cavelerius saccharivorus* and *Blissus insularis* being affiliated with *Burkholderia s.str*. [55]. The monophyly of the two eriococcid symbionts and the long branch separating them from the other *Burkholderia* species, as well as their high degree of synteny, strongly indicate a common ancestry of the *A. aceris* and *G. spuria* symbionts and a shared history of genome erosion that predated the split of the two eriococcid host species. Most of the closest relatives are plant or animal pathogens, suggesting a pathogenic ancestry of the symbionts.

## Discussion

Bacteria of the genus *Burkholderia* show a broad spectrum of interactions with other organisms - from pathogenic to mutualistic [23–25, 28]. Here, we demonstrate that a strain of *Burkholderia* established a nutritional intracellular symbiosis with the two eriococcid species *A. aceris* and *G. spuria*. Our genomic analyses revealed that these symbionts have the smallest and most AT-biased genomes of any *Burkholderia* sequenced so far, indicating genome erosion that is typical for long-term associations. However, despite this limitation and their localization outside of bacteriomes, the *Burkholderia* symbionts in eriococcids fulfill their nutritional function providing host insects with almost all essential amino acids and several B vitamins.

### *Burkholderia*—a new intracellular symbiont of insects

Bacteria from the genus *Burkholderia* are widespread as extracellular symbionts in insects. They usually occur in the midgut crypts (in stinkbugs and bean bugs) or special dorsal organs (in beetles) and fulfill nutritional, detoxifying, or defensive roles [27, 56]. For example, in *Lagria* species, *Burkholderia* produces bioactive secondary metabolites that protect the beetles’ eggs and larvae from microbial infections [27], while in the bean bug *Riptortus pedestris* the symbiont recycles host metabolic wastes [56] and detoxifies pesticides [57]. To our knowledge, eriococcids with *Burkholderia* cells localized within the cytoplasm of fat body cells present the only well-documented example of an intracellular heritable symbiosis with these bacteria in insects [17]. While Ishii et al. [58] detected *Burkholderia* in bacteriome and ovary samples of the leafhopper *Macrosteles striifrons* by PCR-based methods, the exact localization, as well as ultrastructure of these microorganisms, remain unknown due to the lack of microscopic observations. In fact, the authors of the study carefully conclude that *Burkholderia* ‘may be either an environmental contaminant or a gut microbe, but […] the possibility that it may represent a previously unknown type of vertically transmitted *Burkholderia* endosymbiont cannot be ruled out, and deserves future experimental verification’. However, it is worth mentioning that a species of the genus *Mycetohabitants* (*Candidatus* Mycetohabitans vallotii) belonging to *Burkholderia s.l*. has been identified in the cytoplasm of the bacteriocytes of adelgids from the genus *Adelges* [59]. Outside the metazoans, intracellular symbionts representing *Burkholderia s.l*. (formerly classified as *Burkholderia s.str*.) have been detected in the plant pathogenic fungus *Rhizopus* sp. (bacterium *Mycetohabitans*) and in amoebae (bacterium *Paraburkholderia*) [60–63]. In both cases, the host benefits from the bacterial symbiont’s biosynthetic capacities to obtain nutrients. In the soil ameba *Dictyostelium discoideum*, the *Paraburkholderia* symbiont does not provide nutrients directly to the host cell, but it induces co-infections with secondary bacteria that then serve as a food source [64]. By contrast, *Mycetohabitans rhizoxinica* symbionts in the pathogenic fungus *Rhizopus* are responsible for the production of rhizoxin - an antimitotic toxin that kills the host plant of the fungus, allowing the latter to obtain nutrients from the decaying plant [60]. Both above-mentioned symbioses are not completely closed, as these symbionts can be acquired through vertical and horizontal transmission routes [60, 62]. Intracellular localization of horizontally transmitted symbionts requires the machinery for host cell invasion. Concordantly, bacteria that are prone to establish symbiotic interactions with a host usually utilize the infection mechanisms known for bacterial pathogens involving type 2 or type 3 secretion systems (T2SS/T3SS) during the colonization of host cells and tissues. The role of T3SS in establishing an intracellular symbiosis was described previously in *Sodalis*-allied bacteria, which are known as widespread insect symbionts [65, 66]. For example, the T3SS in *Sodalis pierantonius* is likely to mediate infection of midgut epithelial stem cells that develop into adult bacteriomes during the metamorphosis of the cereal weevil *Sitophilus oryzae* [67]. Also, Partida-Martinez and Hertweck [60] showed that *Mycetohabitans* living in the cytoplasm of *Rhizopus* fungi possess T2SS genes that are involved in the secretion of lytic enzymes allowing bacteria to penetrate the fungal cell wall and invade the host cells [67]. Our analyses revealed no evidence for T2SS or T3SS genes in the *Burkholderia* symbionts of *G. spuria* and *A. aceris*, indicating that the *Burkholderia* symbionts in eriococcids permanently adapted to the vertical transmission and intracellular lifestyle.

### *Burkholderia* symbionts in eriococcids show higher nutritional capacity than symbionts of other sap-sucking hemipterans

Obligate symbioses in hemipterans feeding on unbalanced diets are diverse and involve various bacterial taxa [68]. However, all of them show a similar functional pattern— symbionts supplement the host insect with limited essential amino acids and vitamins [4, 68]. While some hemipteran lineages host only one obligate symbiont (for example mealybugs from Phenacoccinae subfamily hosting only *Tremblaya phenacola*, or some aphids harboring *Buchnera aphidicola*), in others the synthesis of amino acids and vitamins is partitioned between two or even more microorganisms with complementary metabolic pathways [4]. This metabolic complementarity between symbionts is common in Auchenorrhyncha, as they usually harbor at least two co-obligatory symbionts [69–71]. Our genomic analyses revealed that *Burkholderia* symbionts in eriococcids lack a co-obligate symbiont and have a higher nutritional capacity in comparison to other hemipteran symbionts, retaining the complete biosynthesis pathways for eight essential amino acids plus precursors for tyrosine (additionally, *Burkholderia* associated with *G. spuria* can synthesize tryptophan, whereas *Burkholderia* in *A. aceris* cannot). However, both symbionts lack a complete gene set essential for the synthesis of methionine. Only the gene *metH* encoding cobalamin-dependent methionine synthases is present in both genomes, whereas the remaining genes *metC*, *metB*, and *metA*, responsible for the transformation of homoserine into homocysteine, are missing. It is possible that *Burkholderia* in eriococcids obtain homocysteine from cysteine through the transsulfuration pathway as they are predicted to synthesize cysteine. The presence of *metH* is not common among bacterial symbionts insects, and most of them are not able to synthesize cobalamin, which is not required by host insects [72]. Therefore, most insect symbionts instead encode *metE* – the cobalamin-independent methionine synthase. The exceptions are *Hodgkinia* and *Evansia*, which, similarly to the eriococcids’ *Burkholderia*, are capable of producing cobalamin and encode *metH* [53, 54]. The lack of some genes involved in methionine biosynthesis is common in symbionts and has been described before in *Buchnera*, *Tremblaya*, *Carsonella*, and *Vidania* [71, 73–75]. Hansen and Moran [73] showed that in aphids, *Buchnera* and its host cooperate in the production of methionine, with products of host genes completing the missing steps in methionine biosynthesis.

The only difference between the genomes of the two *Burkholderia* strains in essential amino acids biosynthesis pathways is related to tryptophan biosynthesis. In contrast to *G. spuria*, in *Burkholderia* of *A. aceris trpC, trpD*, and *trpF* genes were disrupted by frameshift mutations (Fig. S2). This may indicate a recent pseudogenization event or reflect suboptimal expression through transcriptional slippage, as has been observed in other AT-rich endosymbionts [76]. This process would allow for maintaining the functionality of the *trp* operon despite the presence of apparent frameshift mutations, in line with the observation that most plant-feeders including planthoppers, scale insects, and aphids preserve functional tryptophan-biosynthetic pathways [71, 77, 78]. However, we only observe frameshift mutations in the *trp* genes and not in other metabolic pathways, suggesting that the symbionts are indeed in the process of losing a functional tryptophane biosynthetic pathway. Additionally, biosynthesis of tryptophan is one of the most expensive among essential amino acids, and therefore its synthesis is commonly outsourced to co-obligate symbionts, indicating that it is probably one of the pathways most rapidly lost when there is an alternative source for this amino acid [4]. Such potential losses of individual biosynthetic pathways took place in other symbioses as well and are usually presumed to be correlated with switches to diets that contain the missing nutrient [37, 79]. As we did not detect any other microbes associated with *A. aceris* that could supplement the host’s diet with tryptophan, we hypothesize that the insects obtain this amino acids in sufficient amounts from the phloem sap of the host plant *A. platanoides*.

In addition to the pathways for essential amino acid biosynthesis, both symbionts are capable of synthesizing nonessential amino acids, including serine, cysteine, proline, and glycine. As these nutrients can be produced by host insects, the genes involved in their synthesis have not been conserved in highly specialized symbionts like *Sulcia*, *Carsonella* or *Tremblaya*, with the only exception being *Buchnera* of the aphid *Brevicoryne brassicae*, which is predicted to produce cysteine (CP034882). However, some of the additional symbionts that complement the ancient ones, including *Purcelliella* in planthoppers and *Baumannia* in sharpshooters, also encode the complete biosynthetic pathway for cysteine, whereas, in other additional symbionts, only single genes were retained [69, 80]. The presence of complete sets of genes involved in the synthesis of four nonessential amino acids in *Burkholderia* symbiont genomes of ericoccids indicates that these symbiosis are likely younger than other hemipteran symbioses.

Besides amino acids, the *Burkholderia* symbionts of *A. aceris* and *G. spuria* provide cobalamin, riboflavin, thiamine, and folate to the host insect. Such a wide range of B vitamin biosynthetic pathways is unusual among bacterial symbionts of hemipterans, as most of them produce no or only one B vitamin (e.g., cobalamin - *Hodgkinia* and *Evansia*, or riboflavin - *Buchnera*) [53, 54]. However, the record holder in this respect is *Baumannia cicadellinicola* encoding biosynthesis pathways for eight B vitamins, including thiamine, riboflavin, niacin, pantothenic acid, pyridoxine, biotin, and folic acid [69].

### *Burkholderia* replaced the ancestral symbiont of scale insects

In Hemiptera, repeated symbiont replacements have led to a large diversity and variability of symbiotic systems [6, 22, 81]. Concordantly, the evolutionary history of scale insect symbioses is shaped by multiple symbiont replacements [8, 16, 78, 82, 83]. According to Rosenblueth and co-workers [8], Flavobacteria were the likely ancestral symbionts of scale insects since the origin of this insect taxon in the Cretaceous period, and they have been replaced by other microorganisms at least three times in the evolution of Coccomorpha, i.e. in species of the families Putoidae, Pseudococcidae and Dactylopidae. Previous studies on symbionts in the family Eriococcidae [17, 18] revealed that they may host both gammaproteobacterial (*Kotejella* and *Arsenophonus*) and betaproteobacterial (*Burkholderia*) symbionts, suggesting additional replacement events of Flavobacteria in the evolution of scale insects. This hypothesis is supported by the results of our genomic studies of *Burkholderia* symbionts in eriococcids *A. aceris* and *G. spuria*, indicating that these symbioses are very likely younger than some of the other hemipteran symbioses. With sizes of the approx. 900 Kb, the genomes of *Burkholderia* symbionts in examined eriococcids are much smaller than genomes of free-living *Burkholderia* strains (5.5–11.5 Mb) and extracellular *Burkholderia* symbionts in insects (2.3–8.4 Mb) and, in comparison to them, are characterized by much lower GC contents (app. 37%) [32, 33]. On the other hand, they are larger than genomes of other intracellular, heritable hemipteran symbionts, including both ancient ones like *Sulcia* (up to 270 Kb) or *Carsonella* (app. 170 Kb), as well as more recently acquired additional symbionts, e.g., *Purceliella* (app. 480 Kb) or *Baumannia* (app. 680 Kb) [69, 71, 75]. Moreover, their nutritional capacities stand out from other symbionts as they produce almost all essential amino acids, some nonessential ones, and four B vitamins. Thus, taking into account the high degree of similarity between *Burkholderia* strains associated with the two examined eriococcids species and the significant reduction of their genomes that nevertheless retain a broad spectrum of nutritional capacities, we conclude that the symbiosis with *Burkholderia* in Eriococcidae is a result of a symbiont replacement event that occurred before the split of the genera *A. aceris* and *G. spuria*. However, the lack of genomic data for other Eriococcidae species precludes inferences on the distribution and evolution of *Burkholderia* symbionts in this family.

The *Burkholderia* localization provides additional evidence for this symbiosis being younger than other symbioses in scale insects, as they do not exhibit the typical localization in bacteriocytes, but are present in the cytoplasm of fat body cells. Bacteriocyte-localized symbionts are pervasive in Sternorrhyncha and Auchenorrhyncha [9, 70, 71, 81, 84, 85]. The limited data about eriococcids symbioses suggest that these insects ancestrally contained bacteriomes, as the gammaproteobacterial symbionts of *G. brachypodii* - another representative of Eriococcidae family are also bacteriocyte-associated [18]. Thus, the loss of bacteriocytes in the examined eriococcids probably took place upon the acquisition of the *Burkholderia* symbiont. A similar loss of symbiont-containing organs has been observed in some cicadas, Deltocephalinae leafhoppers, and planthoppers, in which one or both of the bacteriome-associated symbionts (*Hodgkinia* in cicadas, *Nasuia* in Deltocephalinae leafhoppers, both *Vidania* and *Sulcia* or only *Sulcia* in planthoppers) have been replaced with a fat body-localized *Ophiocordyceps* fungus that evolved from a pathogenic ancestor [22, 81]. It is possible that *Burkholderia* symbionts of eriococcids have experienced a similar evolutionary history, as our phylogenomic analysis indicates that they fall into the (mostly) pathogenic *Burkholderia s.str*. clade. Symbiont replacements are common among hemipterans [78, 86–89], and they may allow the hosts to escape the rabbit hole of degenerative symbiont genome evolution [87]. The newly acquired symbionts (e.g. gammaproteobacteria *Arsenophonus* and *Sodalis* or *Ophiocordyceps*-allied fungi) initially possess large genomes, which likely makes them more effective in their nutritional functions [90]. However, after the replacement, functional convergence of symbiont-encoded traits and genome erosion involving reduction of genome size and AT bias usually occur [90].

The change in the symbiont localization from bacteriocytes into fat body cells likely influenced the communication and integration of host-symbiont interaction. As opposed to bacteriomes that are specialized in maintaining and regulating symbionts, fat body cells serve additional purposes, e.g., the production of hydrocarbons and fatty acids, vitellogenins, as well as immune effectors [91]. Therefore, the lack of bacteriocytes and localization in fat body tissue probably affected the evolution of the symbiosis and the regulation of the symbionts by the host. The key to testing hypotheses concerning the evolution of symbiosis will be the comparative analysis of symbioses involving taxonomically different symbiotic microorganisms showing the same localization patterns: *Burkholderia* in Eriococcidae, *Ophiocordyceps* in cicadas, planthoppers and aphids, and *Blattabacterium* in cockroaches.

### *‘Candidatus* Burkholderia endosymbiotica sp. nov.’

As *Burkholderia* symbionts of *A. aceris* and *G. spuria* represent the only insect-associated intracellularly localized *Burkholderia* strains, we propose to name them ‘*Candidatus* Burkholderia endosymbiotica *sp. nov*.’, and denote the host affiliation with AACE and GSPU, respectively (i.e., ‘*Candidatus* Burkholderia endosymbiotica AACE’, and ‘*Candidatus* Burkholderia endosymbiotica GSPU’). Phylogenetic position: *Pseudomonadota*, *Burkholderiales, Burkholderiaceae*; Gram-negative bacteria, uncultured obligate intracellular symbionts of eriococcids: *A. aceris* and *G. spuria* (Hemiptera, Coccomorpha: Eriococcidae); shape and size: rod-shaped bacteria, approximately 2 µm in length and 1 µm in diameter; localization: cytoplasm of fat body cells; role in host biology: nutritional symbionts (providing essential amino acids and B vitamins); inherited vertically (transovarially) between generations; described based on genomes deposited in the NCBI database under Bioproject PRJNA981321 (other sequences deposited in the NCBI belonging to this species: KR904884 and KR904885).

## Conclusions

Symbiont acquisitions and replacements represent important driving forces of insects’ evolution and diversification. Establishing symbiosis with ‘new’ microorganisms of large biosynthetic capacities may drastically increase the fitness of host insects and allow them to adapt to novel ecological niches. The obligatory, heritable, nutritional symbiosis between eriococcids and *Burkholderia* offers new insights into the diversity of beneficial intracellular symbionts that can be recruited by insects and provide additional evidence that host-beneficial bacteria often have a pathogenic origin. Observed changes in symbiont localization, together with functional convergence, in the future may provide valuable insights into the flexibility but also constraints on symbiont metabolism and host control.

### Supplementary information

Sypplementary information

## Data Availability

Sequence data have been deposited in GenBank under Bioproject PRJNA981321. Raw data have been deposited on the MPG Edmond repository (https://edmond.mpdl.mpg.de/) under the link 10.17617/3.3CEYBM.
