# Supplementary material for: Nutrient supplementation by genome-eroded Burkholderia symbionts of scale insects
Source: ISME J. 2023 Oct 13;17(12):2221–31. doi: 10.1038/s41396-023-01528-4 (PMC10689751; doi:10.1038/s41396-023-01528-4)
Supplement: Supplementary file 1 — Sypplementary information [file 41396_2023_1528_MOESM1_ESM.docx]

**Nutrient supplementation by genome-eroded *Burkholderia* symbionts of scale insects**

Anna Michalik, Eugen Bauer, Teresa Szklarzewicz, Martin Kaltenpoth

**SUPPLEMENTARY INFORMATION**


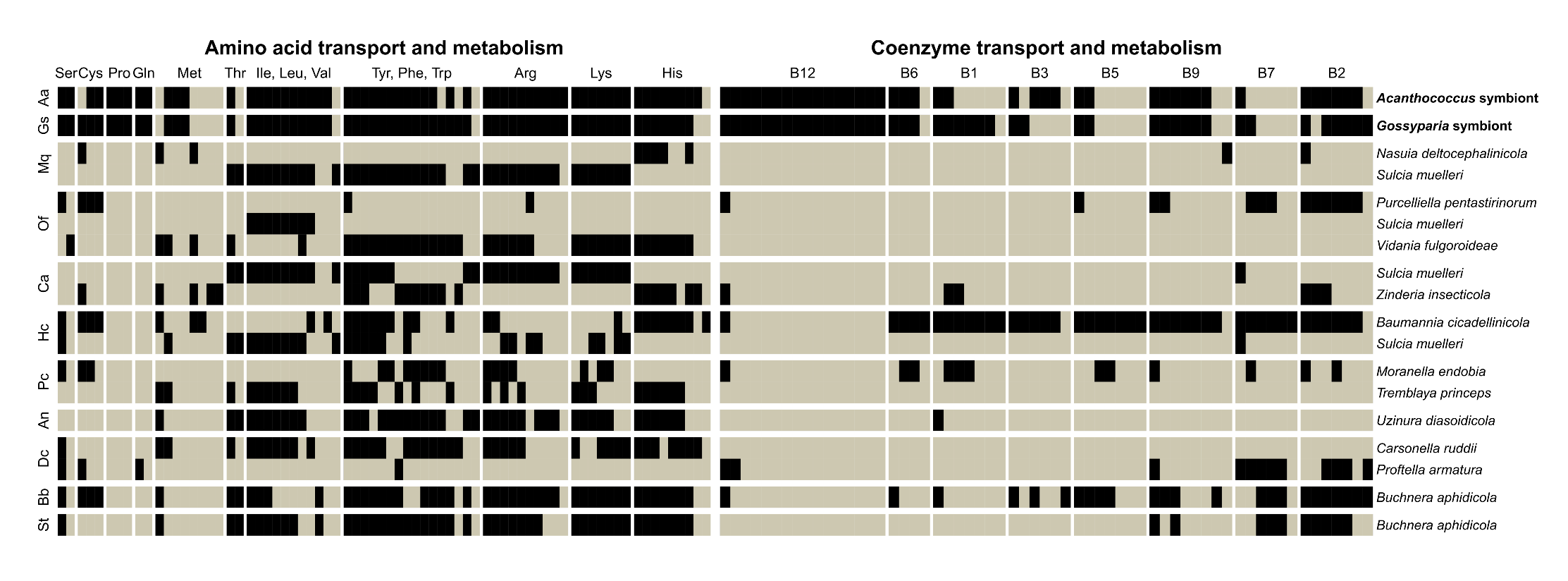


**Figure S1.** Comparison of biosynthetic capabilities of *Burkholderia* symbionts of Eriococcidae and other hemipterans’ obligate and co-obligate symbionts.


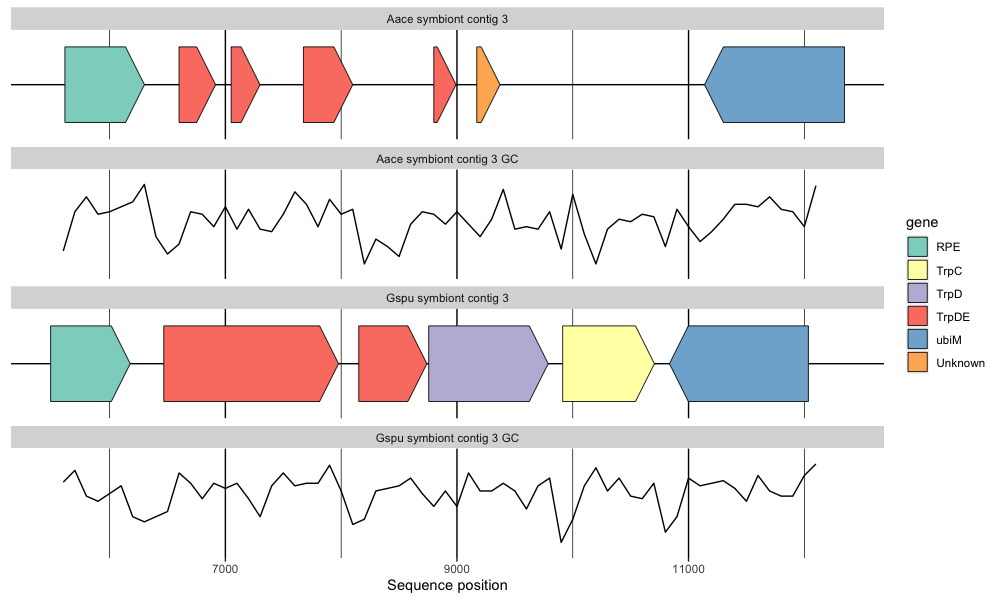


**Figure S2.** Comparison of the operon encoding tryptophan biosynthesis genes of *Burkholderia* symbionts of Eriococcidae. Sequence positions are in base pairs and the relative GC content was calculated with a sliding window of 100 base pairs.
